# Color image segmentation using adaptive hierarchical-histogram thresholding

**DOI:** 10.1371/journal.pone.0226345

**Published:** 2020-01-10

**Authors:** Min Li, Lei Wang, Shaobo Deng, Chunhua Zhou

**Affiliations:** 1 Nanchang Institute of Technology, Nanchang, Jiangxi, PR China; 2 Jiangxi Province Key Laboratory of Water Information Cooperative Sensing and Intelligent Processing, Nanchang, Jiangxi, PR China; 3 School of Life Sciences, Nanchang University, Nanchang, Jiangxi, PR China; Beijing University of Technology, CHINA

## Abstract

Histogram-based thresholding is one of the widely applied techniques for conducting color image segmentation. The key to such techniques is the selection of a set of thresholds that can discriminate objects and background pixels. Many thresholding techniques have been proposed that use the shape information of histograms and identify the optimum thresholds at valleys. In this work, we introduce the novel concept of a hierarchical-histogram, which corresponds to a multigranularity abstraction of the color image. Based on this, we present a new histogram thresholding—Adaptive Hierarchical-Histogram Thresholding (AHHT) algorithm, which can adaptively identify the thresholds from valleys. The experimental results have demonstrated that the AHHT algorithm can obtain better segmentation results compared with the histon-based and the roughness-index-based techniques with drastically reduced time complexity.

## Introduction

Image segmentation plays a crucial role in the areas of image analysis, pattern recognition and computer vision-related applications. In segmentation, an image is partitioned into different nonoverlapping regions that are homogenous with respect to certain properties, such as color information, edges, and texture [1, 2]. Although many techniques for image segmentation have been proposed, it is still a very challenging research topic due to the variety and complexity of images. Moreover, color images can provide richer information than grayscale images, and natural color image segmentation is increasingly paid more attention by scholars.

Generally, image segmentation routines are divided into histogram-based approaches [3–5], edge detection approaches [6, 7], region-based approaches [8, 9], clustering approaches [10–14] and combinations of several approaches [15–17]. Although a larger number of segmentation algorithms have been developed, each has its own applicability and limitations. The properties of these techniques have been discussed in Ref [18].

One of the most widely applied techniques for image segmentation is histogram-based thresholding, which assumes that homogeneous objects in the image manifest themselves as clusters. The key to the histogram-based technique is the selection of a set of thresholds that can discriminate objects and background pixels. Numerous histogram-based thresholding methods have been proposed over the years. These methods can be broadly classified into two categories. The first category contains thresholding techniques that determine the optimal thresholds by optimizing a certain objective function [19–25]. Among these thresholding techniques, entropy-based approaches are the most popular, and many algorithms have been proposed in this direction. Examples of these include Shannon Entropy, Renyi′s entropy [24,26], entropic correlation [5], and cross entropy [20]. However, the main problem associated with these algorithms is their large time complexity. For the multilevel thresholding problem in Minimum Cross Entropy Thresholding [25,27], the time complexity is *O*(*mL*^*m*+1^), where *m* represents the number of threshold values and *L* indicates the number of gray levels. The second category contains approaches that determine the optimal thresholds by utilizing shape information of the histogram of a given image. The rationale for threshold determination implicitly relies on the assumption that the intensities of pixels, or data in a more general setting, should be similar within the same objects and different between different objects [16]. In this manner, the intensity-level histogram values of each object could appear as a bell-shaped mode [19]. The peak of the bell-shaped region and its adjacent position intensity correspond to the main-body pixels of the object, while the boundary of the bell-shaped region corresponds to the edge pixels of the object. Therefore, the peaks and valleys in the histogram are used to locate the clusters in the image, and the optimum thresholds must be located in the valley regions. For example, Rosenfeld *et al*. investigated histogram concavity analysis as an approach for threshold selection [28]. Lim and Lee presented a valley-seeking approach that smoothes the histogram and detects the valleys as thresholds by calculating the derivatives of the smoothed histogram [29]. Because the histogram only includes the information of intensity levels, these methods do not consider the spatial correlation of the same or similarly valued elements. To overcome this drawback, some variations of the histogram are presented. Mohabey and Ray [30, 31] utilized rough set theory [32] to construct the concept of a histon. Different from a histogram, each bin of a histon is the pixel scale belonging to the corresponding intensity with uncertainty [2]. With the aid of rough set theory, the histogram and histon can be respectively considered as the lower and upper approximations. Mushrif and Ray then proposed using the roughness measure at every intensity level to extract the homogeneous regions of a color image [33]. For some images, however, it is difficult to obtain the significant peaks and valleys of the roughness measure; Xie *et al*. used local polynomial regression to smooth the histogram and histon and then calculated the roughness measure, which enabled their approach to find the real peaks and valleys more easily [34].

Similar to the histogram, both the histon and roughness indexes provide the global information of homogeneous regions in the image, and every peak and its adjacent position represent a homogeneous region. Theory analysis shows that the histon pays little attention to the small homogenous regions, and the roughness index can effectively indicate the region homogeneity degree and avoid the disturbance of imbalanced color distribution. As two variants of the histogram, the histon and roughness index were demonstrated to achieve better segmentation results. Both histon-based and roughness-index-based algorithms, however, need to calculate the color difference between every pixel and its neighborhood, which means both require significant time. The steps of the above techniques involve some smoothing of the histogram (histon or roughness-index) data, searching for significant modes, and placing thresholds at the minima between them.

In this paper, we propose an original segmentation scheme named *AHHT* (Adaptive Hierarchical-Histogram Thresholding), which uses a structure called the *hierarchical-histogram* to adaptively identify the thresholds at valleys for thresholding. A *hierarchical-histogram* includes a group of histograms that corresponds to a multigranularity abstraction of the image. The lower the histogram is in the *hierarchical-histogram*, the more elaborate the details of the image it pertains to are. The role of the prior-level histogram in the *hierarchical-histogram* is generating for the next-level histogram, and the top-level histogram is applied to segment the image. To verify the effectiveness of *AHHT*, experiments are performed on the Berkeley Segmentation Data Set and Benchmark, and a comparison with the histon-based technique and roughness-index-based technique is made in terms of both visual and quantitative evaluations.

This paper is organized as follows. Section 2 reviews the related work. Section 3.1 describes the main idea of the proposed *AHHT* algorithm. Section 3.2 presents the *AHHT* algorithm in detail. Section 3.3 analyzes the complexity of the *AHHT* algorithm. Section 4 analyzes the experimental results. Section 5 concludes the paper.

## Related work

*RGB* is the most commonly used model for the television systems and pictures acquired by digital cameras. As discussed in other related works, this paper also focuses on color image segmentation in the *RGB* color space. Consider *I* to be an *RGB* image of size *M* × *N*, consisting of three primary color components: red *R*, green *G*, and blue *B*. The classic histogram of the image for each color component is defined as

$$h_{i}(l)=\sum_{m=1}^{M}\sum_{n=1}^{N}\delta (I(m,n,i)-l),\;for\;0\leq l\leq L-1\;and\;i\in \left\{R,G,B\right\},$$ (1)

where δ(x)={1,x=00,x≠0 is the indicator function and *L* is the intensity scale in each of the color components. The value *h*_*i*_(*l*) is the number of pixels having intensity *l* in color component *i*.

Let *c*_1_ and *c*_2_ be color vectors in the *RGB* color space. The Euclidean distance between the two vectors is given by

$$d(c_{1},c_{2})=\sqrt{\sum_{i\in \left\{R,G,B\right\}}{\left(c_{1}(i)-c_{2}(i)\right)}^{2}}.$$ (2)

For a *P* × *Q* neighborhood around a pixel *I*(*m*, *n*), the color difference between *I*(*m*, *n*) and its surrounding pixels in neighborhood is defined as [33]:

$$d_{T}(m,n)=\sum_{p\in P}\sum_{q\in Q}d(I(m,n),I(p,q)).$$ (3)

If the color difference *d*_*T*_(*m*, *n*) is less than a threshold *T*_0_, the surrounding pixels in neighborhood fall in the sphere of a similar color. For an *RGB* image *I* of size *M* × *N*, a matrix *I*′ of size *M* × *N* is defined such that an element *I*′(*m*, *n*) is given by

I′(m,n)={1,dT(m,n)<T00,otherwise. (4)

Then, the histon is defined as follows [31]:

$$h_{i}^{\prime }(l)=\sum_{m=1}^{M}\sum_{n=1}^{N}\left(1+I(m,n))\delta (I(m,n,i)-l\right),\;for\;0\leq l\leq L-1\;and\;i\in \left\{R,G,B\right\}.$$ (5)

The histogram and the histon can be associated with the concept of approximation space in rough set theory [32,35]. For intensity class *l*, the value of *h*_*i*_(*l*) is the number of pixels that have intensity value *l* and therefore can be viewed as the lower approximation, and the value of $h_{i}^{\prime }(l)$ can be considered as the upper approximation. Mushrif and Ray then proposed the roughness measure as follows [33].

$$\rho_{i}(l)=1-\frac{h_{i}(l)}{h_{i}^{\prime }(l)},\;for\;0\leq l\leq L-1\;and\;i\in \left\{R,G,B\right\}.$$ (6)

**Fig 1:**
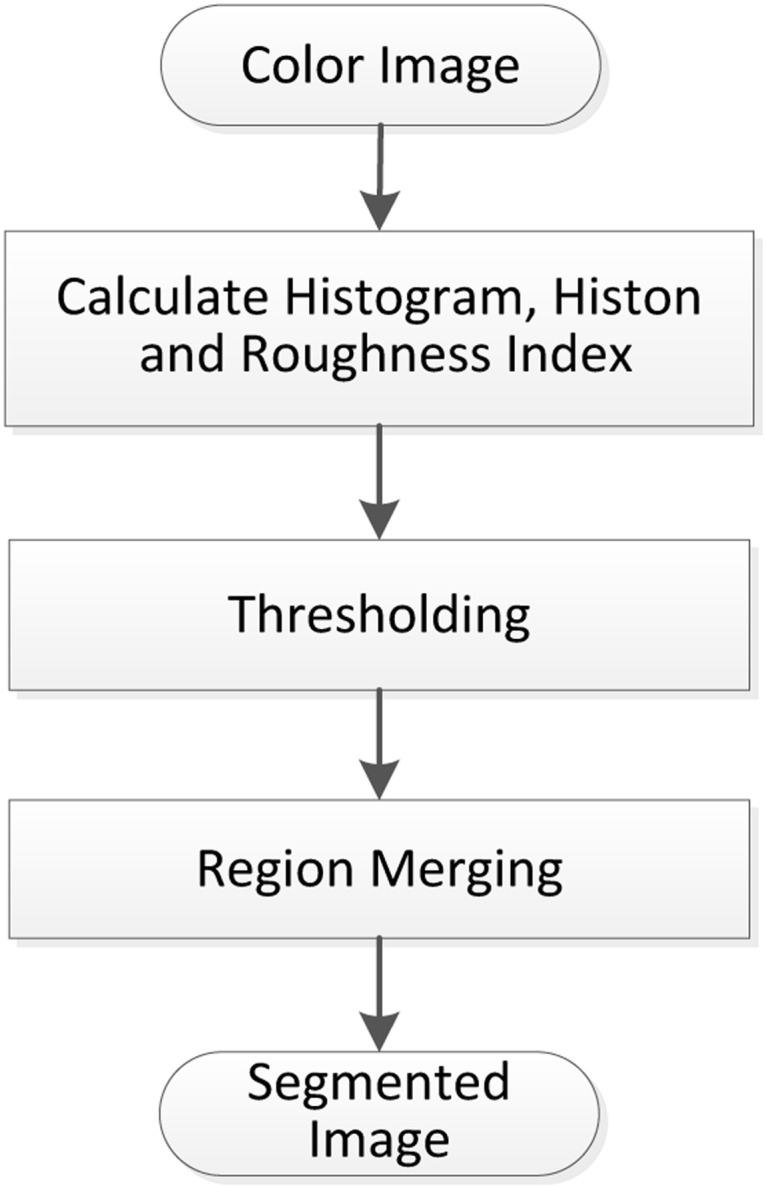
Flowchart of the histogram-based technique.

Like the histogram, the histon and the roughness index for all intensity values also give the global information of homogeneous regions in the image, and every peak and its adjacent position represent a homogeneous region. Therefore, the histon and the roughness index are two variations of the histogram. The histogram, the histon and the roughness index are collectively called histogram-based techniques in this paper. The segmentation process of such histogram-based techniques is divided into three stages [33], as shown in Fig 1.

We take the roughness-index-based [33] technique for example to illustrate the flowchart of Fig 1. First, the roughness index for each plane of *R*, *G* and *B* of the image is calculated. Then, two criteria are used to obtain the significant peaks of the roughness indexes: (1) the height of the peak is greater than 20% of the average value of all peaks; and (2) the distance between two peaks is greater than 10. After the significant peaks are selected, the thresholds are identified at the minima between every two adjacent significant peaks. Second, all selected thresholds are applied to split the image into multiple clusters. The color representing each cluster is obtained by averaging all the pixels within the cluster. At this point, the initial segmentation is completed. Generally, this process usually results in over-segmentation. Lastly, the *Region-Merging* process uses the algorithm proposed by Cheng et al. [36] to deal with small regions and similar regions. Concretely, the following two steps are carried out. (1) The clusters with pixels less than a predefined threshold *T*_*n*_ are merged with the nearest clusters. (2) Two closest clusters are combined to form a single cluster if the distance between the two clusters is less than a predefined threshold *T*_*d*_.

The basic thresholding procedure consists of analysis of an image histogram and subsequent threshold selection from the values located in the valleys between peaks. However, the determination of peaks and valleys in a multimodal histogram is a nontrivial problem. In general, there are many local peaks and local valleys in the histogram of each color space of an *RGB* color image. The steps of the above techniques involve some smoothing of the histogram data, searching for significant peaks, and then identification of thresholds at the minima between two adjacent significant peaks. This means that the selection of significant peaks will be used to determine the thresholds, which consequently determine the final segmentation result of the image. As such, the above histogram-based thresholding techniques mainly focus on how to identify the significant peaks in the histogram, and then identify the valleys for thresholding. As two variants of the classic histogram, the histon and roughness index were demonstrated to achieve better segmentation results. Both histon-based and roughness-index-based algorithms, however, need to calculate the distance between every pixel and its neighborhood, which means that significant time is required to calculate the histograms. In addition, as mentioned above, both algorithms also need to determine the significant peaks to identify the thresholds. Moreover, it is difficult for these techniques to find the exact threshold point if the valley is flat.

## Color image segmentation based on adaptive hierarchical-histogram thresholding

In this section, we propose a segmentation technique that uses a hierarchy structure of histograms to adaptively obtain the thresholds for color image segmentation. Our method does not need to find the significant peaks, it can adaptively identify the thresholds from valleys, and it has high efficiency.

### Main idea of adaptive hierarchical-histogram thresholding

Based on experiments performed on hundreds of *RGB* color images, we found that each image yields dozens of local valleys and local peaks in each histogram of the *R*, *G*, and *B* planes. As presented above, in the histogram, the peak of the bell-shaped region and its adjacent position intensity correspond to the main-body pixels of the object, while the boundary of the bell-shaped region corresponds to the edge pixels of the object. Therefore, in the histogram, the intensities between every pair of adjacent local valleys correspond to a small breadth bell-shaped region. All pixels ranged in a small bell-shaped region can also be regarded as a small homogeneous region. If we use all local valleys in the histogram of each color plane to segment an image, the image will be divided into a mass of small homogeneous regions. The colors of these small homogeneous regions will very close to the corresponding colors in the original image because such segmentations are overelaborate. Although such segmentation is exquisite, the segmented image can be viewed as an abstract version of the original image. For the original image, we noticed that a more abstract version with a relatively small number of homogeneous regions can be generated based on the segmented image. This finding is what inspired us to propose the *AHHT* algorithm for color image segmentation. The main idea of *AHHT* is to build a group of hierarchical histograms that corresponds to a multigranularity abstraction of the original image.

For each different color space, *AHHT* adopts a bottom-up approach to generate a group of histograms that form a hierarchy graph, and the obtained top-level histograms will be applied to segment the image. The rough process of *AHHT* is as follows. In each plane of *R*, *G*, and *B*, according to Eq 1, the histogram is calculated as the first (bottom)-level histogram. From the first-level histogram, each small bell-shaped region is merged into a bin expressed by the count (the number of pixels within the intensity range of the small bell-shaped region) and the weighted average intensity (the average intensity of all pixels within the small bell-shaped region), and then the second-level histogram is obtained. Next, similar action is applied to generate the third-level histogram, that is, from the second-level histogram, each bell-shaped region is merged into a bin expressed by the count and the weighted average intensity of all pixels within the bell-shaped region. Such process continues until the last-generated histogram has no valleys or the difference of every adjacent pair of bins is larger than a threshold *w*. Obviously, each bin of the top-level histogram corresponds to a group of pixels in the image. All bins′ information in the top-level histogram in each plane of *R*, *G*, and *B* is applied to split the image into multiple clusters. The color representing each cluster is obtained by averaging all the pixels within the cluster. At this point, the initial segmentation is completed. In the process of *Region-Merging*, the *AHHT* algorithm adopts an approach identical to that used in the roughness-index-based algorithm.

In each color plane of *R*, *G*, and *B*, *AHHT* generates a group of histograms in a hierarchical fashion. Hereafter, such a group of histograms is called a *hierarchical-histogram*. The lower a histogram is in the *hierarchical-histogram*, the more elaborate the details of the image it encodes are. The role of the prior-level histogram is generating for the next-level histogram. The experiments performed on hundreds of color images show that *AHHT* commonly generates four to five histograms for each color plane of the image. The *AHHT* algorithm segmentation of the image is based on the top-level histograms.

**Fig 2:**
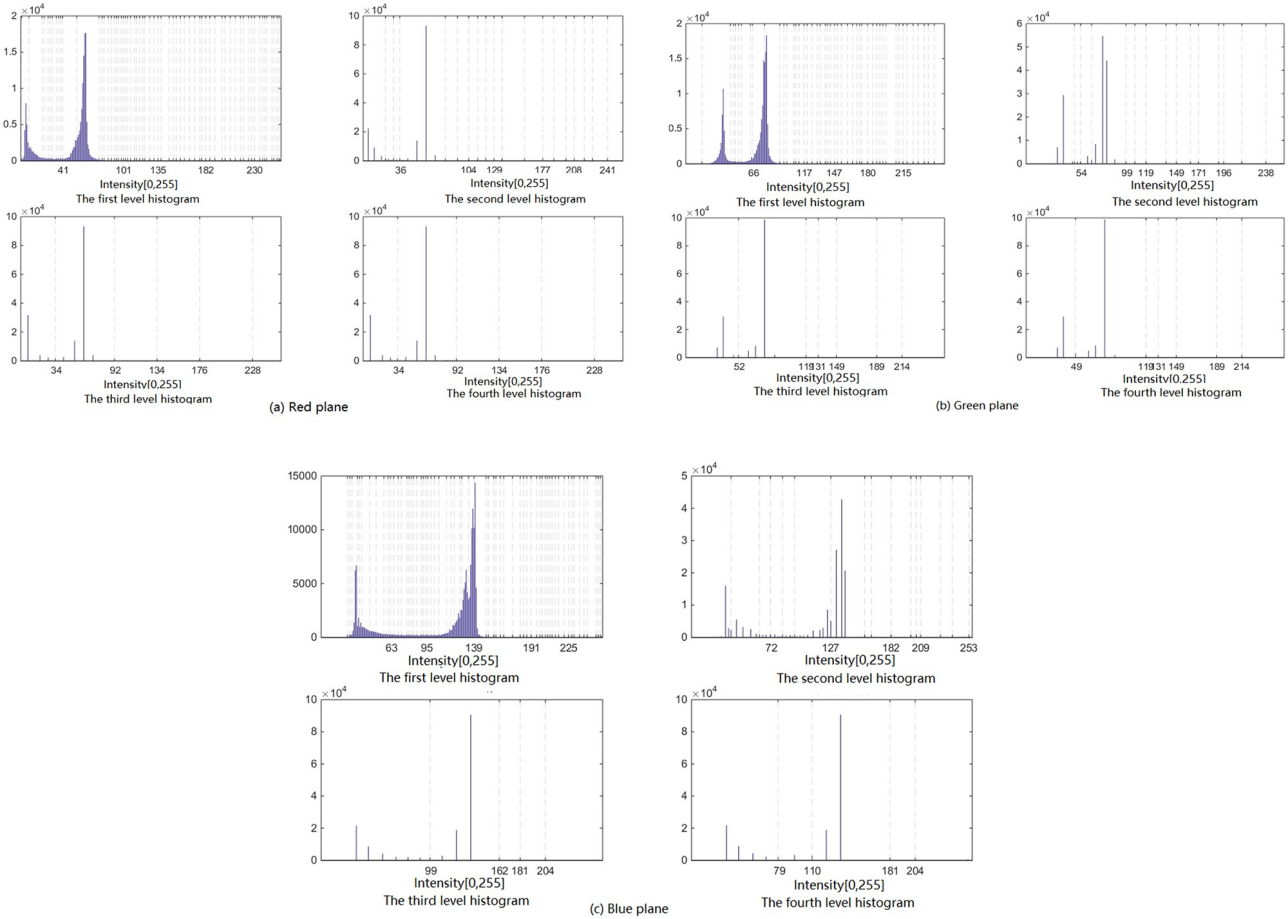
Hierarchical-histogram of each plane of R, G, and B of the image Moon obtained by AHHT.

**Fig 3:**
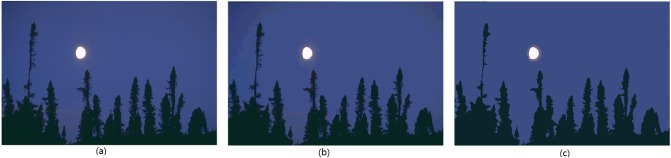
The image Moon: (a) original image, (b) initial segmented result (225 colors), (c) final segmented result (4 colors).

The *hierarchical-histogram* for each plane of *R*, *G*, and *B* of the image *Moon*, generated by the *AHHT* algorithm, are shown in Fig 2(a)–2(c), respectively. In the experiment, the parameter *w* = 20, which means that the difference of every adjacent pair of bins in the histogram is larger than 20, and the top-level histogram is generated. As shown in Fig 2, *AHHT* generates four histograms for each color plane. The first-level histogram is generated from the original image *Moon*, and the next-level histogram is generated from the prior-level histogram. In each histogram of Fig 2, each dashed line marks a valley′s position. From Fig 2, we can see that the first-level histogram (of each plane of *R*, *G*, and *B*) has many local valleys, which means that there are many small bell-shaped regions in the first-level histogram. Each small bell-shaped region in the first-level histogram is expressed by a bin in the second-level histogram, and so on. The fourth-level (top-level) histogram of each color plane is applied to segment the image. In the *Region-Merging* process of this experiment, the regions with fewer than 0.1% of the pixels are merged with the nearest region, and two regions with a distance of less than 70 are combined to form a single region. Fig 3(b) and 3(c) show the initial segmented result and the final segmented result of the image *Moon*, respectively.

### Algorithm

In a histogram, a valley corresponds to a local minimum, which is present near a local lowest point or a local lowest horizontal line. All valleys in the histogram of a color plane can be identified by the following rule.

$$\begin{array}{l} if\;h_{i}\left(l\right)<h_{i}\left(l-1\right)\;\&\;h_{i}\left(l\right)<h_{i}\left(l+1\right)\\ \;\text{then}(l\;is\;valley)\\ if\;h_{i}\left(l\right)<h_{i}\left(l-1\right)\;\&\;h_{i}\left(l\right)=h_{i}\left(l+1\right)=\cdots =h_{i}\left(l+k\right)\;\&\;h_{i}\left(l+k\right)<h_{i}\left(l+k+1\right)\\ \;\text{then}(l+\left\lfloor k/2\right\rfloor is\;valley,\;f\text{or}\;0\leq l\leq L-1\;\text{and}\;i\in \left\{R,G,B\right\}. \end{array}$$ (7)

In our program, a bin in the histogram is expressed as a triple of [*h*(*l*), *l*, *lR*], where *l*(0 ≤ *l* ≤ *L* − 1) is the intensity, *h*(*l*) is the number of image pixels having intensity *l*, and *lR* is the right endpoint′s intensity of the bin. *H* is an array of bins in ascending order according to intensity, which means that *H* corresponds to a histogram. The *m*th and (*m* + 1)th elements of *H* are *H*[*m*] = [*h*(*l*_*m*_), *l*_*m*_, *l*_*m*_*R*] and *H*[*m* + 1] = [*h*(*l*_*m*+1_), *l*_*m*+1,_ *l*_*m*+1_*R*], respectively, where *l*_*m*_ < *l*_*m*+1_ holds. The first-level histogram *H* is generated from the original image. For every bin [*h*(*l*), *l*, *LR*] of the first-level histogram, *lR* = *l* holds. According to Eq 7, a function named *GetValleys*(*H*) is used to find all valleys (sorted in ascending order) from a histogram *H*. The details of the function are left out in order to keep the paper reasonably concise. If *H* is generated from the original image, which means that *H* is the first-level histogram, then *GetValleys*(*H*) can find all local valleys that will be used to generate the second-level histogram. If *H* is the *k*th (*k >* 1)-level histogram, then the result of *GetValleys*(*H*) will be used to generate the (*k* + 1)th-level histogram. **Function 1 –***GetNextHist* is used to generate the next-level histogram of *H*, and the pseudocode is as follows.

**Function 1.** *GetNextHist*(*H*, *valleys*, *w*).

Input: a histogram *H* and its v*alleys*; bin merge threshold *w*;

Output: *H*′, which is the next-level histogram of *H*;

(1) *H*′ = ∅; //initialize the result

(2) *left* = 1; *right* = 1; // left-end and right-end intensities of a bell-shaped region

(3) *bins* = []; //store bins of a bell-shaped region

(4) *for r* = 1 *to length*(*valleys*)

(5)  *for m* = *left to length*(*H*)//find the bin for which the intensity equals *valleys*[*r*]

(6)   [*h*(*l*_*m*_), *l*_*m*,_ *l*_*m*_*R*] = *H*[*m*]; //fetch the *mth* bin of *H*

(7)   *if* *l_m_* ⩵ *valleys*[*r*] *then*

(8)    {*right* = *m*; *break*;}

(9)  *bins* = *GetMergeBin*(*H*, *left*, *right*, *w*); //generate bins for the bell-shaped region

(10)  *H′* = *H′* ∪ *bins*;// Append every *bin* of *bins* to *H*′

(11)  *left* = *right* + 1

(12) *bins* = *GetMergeBin*(*H*, *left*, *length*(*H*), *w*); //generate bins for last bell-shaped region

(13) *H′* = *H′* ∪ *bins*;

(14) *return H′*;

In lines 9 and 12, a function named *GetMergeBin* returns bin or a group of bins for a bell-shaped region. In line 9, the parameters *left* and *right* of *GetMergeBin* are the left-end index and right-end index of a bell-shaped region in the histogram *H*. If the difference between the right-end intensity and left-end intensity is less than *w*, then *GetMergeBin* returns a bin corresponding to the bell-shaped region; otherwise, at most ⌊(*l*_*right*_ − *l*_*left*_)/*w*⌋ +1 bins will be returned. For simplicity, the pseudocode of *GetMergeBin* is omitted. Note that the function *GetMergeBin* only merges adjacent bins within a bell-shaped region. This mechanism makes the next-level histogram match the original intensity distribution of the image well.

On the basis of the above functions, Function 2 –*GetAHH* (Get Adaptive Hierarchical-Histograms) is used to generate a *hierarchical-histogram* for each plane of *R*, *G*, and *B* of the image, and the pseudocode is as follows.

**Function 2.** *GetAHH*(*I*, *w*).

Input: an *RGB* color image *I*; bin merge threshold *w*;

Output: *hierarchical-histogram* for each plane of *R*, *G*, and *B* of the image;

(1) for each color plane *i* ∈ {*R*, *G*, *B*} of image *I*

(2)  *Hists*_*i*_ = ∅; //store a hierarchical-histogram;

(3)  Calculate *w*_*i*_ according to Eq 8;

(4)  Generated the first-level histogram *H*_*i*_;

(5)  Append *H*_*i*_ to *Hists*_*i*_;

(6)  *valleys* = *Getvalleys*(*H*_*i*_);

(7)  $H_{i}^{\prime }=GetNextHist\left(H,valleys,w_{i}\right)$

(8)  while $H_{i}\prime \neq H$ do

(9)    Append $H_{i}^{\prime }$ to *Hists*_*i*_;

(10)   $H_{i}=H_{i}^{\prime }$;

(11)   *valleys* = *Getvalleys*(*H*_*i*_);

(12)   $H_{i}^{\prime }=GetNextHist\left(H,valleys,w_{i}\right)$

(13) return *Hists*_*i*_ for each *i* ∈ {*R*, *G*, *B*}

For each color plane of the image, a *hierarchical-histogram* is generated starting with the first-level histogram, and the next-level histograms are iteratively generated until the new-level histogram has no change.

For an *RGB* color image, there are different widths of valid intensity between each plane of *R*, *G*, and *B*. Taking Fig 2‘s image of *Moon* as an example, the (first-level) histogram of the Blue plane has a wide distribution of valid intensity, and the (first-level) histogram of the Green plane has a relatively narrow distribution of valid intensity. Therefore, different threshold values *w* should be set for different color planes. A reasonable threshold *w* should be given a relatively large value for a color plane with a wide width of valid intensity and a relatively smaller value for a color plane with a narrow width of valid intensity. For different color planes, the threshold *w*_*i*_ can be calculated as follows.

$$\begin{array}{l} w_{i}=w\times \frac{SPAN_{i}}{L},\;\text{for}\;i\in \left\{R,G,B\right\},\\ SPAN_{i}=argmax\left({}_{0\leq l\leq L-1}h_{i}\left(l\right)\neq 0\right)-argmin{(}_{0\leq l\leq L-1}h_{i}\left(l\right)\neq 0). \end{array}$$ (8)

According to Eq 8, *w*_*i*_ is calculated as the value of *w* multiplied by the scale factor $\frac{{SPAN}_{i}}{L}$, where *SPAN*_*i*_ is the difference between the max valid intensity and min valid intensity of the color plane *i*. In this manner, a relatively larger threshold *w*_*i*_ is applied for a color plane with a wide width of intensity. However, for some images, there are only a very small number of pixels distributed at lower intensities or higher intensities of a color plane, which makes a relatively larger threshold *w*_*i*_ be applied to the color plane. To avoid noise trouble, we use the *SPAN*_*i*_ of Formula (9) to replace the *SPAN*_*i*_ of Formula (8).

$${SPAN}_{i}={\text{argmax}}_{0\leq l\leq L-1}\left(\frac{\sum_{l}^{L-1}h_{i}(l)}{n}>0.01\right)-{\text{argmin}}_{0\leq l\leq L-1}\left(\frac{\sum_{0}^{l}h_{i}(l)}{n}>0.01\right)$$ (9)

In expression 9, the threshold value of 0.01 means the *SPAN*_*i*_ is the difference between the max valid intensity and min valid intensity, excluding the top 1 percent and bottom 1 percent of pixels, which improves the robustness of the calculation of *SPAN*_*i*_.

Once the *hierarchical-histograms* for each *R*, *G*, and *B* plane are generated, the top-level histograms in the *hierarchical-histograms* are used to segment the image. Concretely, for a color plane *i*, every pixel with an original intensity value range of [*l*_*m*−1_*R*, *l*_*m*_*R*]) is set to the intensity value of *l*_*m*_, where *l*_*m*−1_*R*, *l*_*m*_*R* and *l*_*m*_ come from the (*m* − 1)th and *m*th elements (bins) in the top-level histogram *H*_*i*_, that is, *H*[*m*] = [*h*(*l*_*m*_), *l*_*m*_, *l*_*m*_*R*] and *H*[*m* + 1] = [*h*(*l*_*m*−1_), *l*_*m*−1_, *l*_*m*−1_*R*]. After such process, the initially segmentation is completed. It is pretty remarkable that the obtained top-level histograms correspond to the histogram of each *R*, *G*, and *B* plane of the segmented image. On the basis of the above, *AHHT* (Adaptive Hierarchical-Histogram Thresholding) algorithm for color image segmentation is described as follows.

**Algorithm 1.** *AHHT*(Adaptive Hierarchical-Histogram Thresholding)

Input: an *RGB* color image; bin merge threshold *w*; small region threshold *T*_*n*_; distance *T*_*d*_ for merging close regions;

Output: the segmented image;

Step 1: Calculate the *hierarchical-histogram* for each *R*, *G*, and *B* plane of the image by calling Function 2 –*GetAhh*;

Step 2: Segment the image by using the top-level histograms obtained by Step 1;

Step 3: Merge small regions and close regions.

The *AHHT* algorithm has three main advantages: (1) *AHHT* adopts a bottom-up strategy to build the structure of the *hierarchical-histogram*, which can adaptively identify the thresholds from valleys; (2) In the process of identifying the thresholds, *AHHT* does not need to determine peaks, and only one parameter, *w*, is involved; and (3) *AHHT* finds the thresholds with high efficiency.

### Complexity analysis

The computational complexity of the *AHHT* algorithm is analyzed as follows. An *RGB* color image *I* with n pixels and an intensity scale *L* for each color space is given. The total computation time includes that consumed in each of three major steps.

In the first step, the *hierarchical-histogram* for each color plane is computed. The complexity of generating all three first-level histograms is *O*(3*n*). The complexity of generating all three second-level histograms is *O*(3*L*). The complexity of generating all three *m*th-level histograms is *O*(3*L*_*m*_), where *L*_*m*_ is the average number of bins in the three (*m* − 1)th-level histograms. Because a *hierarchical-histogram* only includes a limited number of histograms, the time required to generate the first-level histograms is far greater than the rest of the time required to generate the others. The complexity of step 1 can thus be considered as *O*(3*n*). In the second step, every pixel is distributed into the corresponding bin and assigned the intensity value of the bin by using the three top-level histograms. It is given that the number of bins in every top-level histogram is *k*. This process requires approximately 3*kn* operations, and the complexity of step 2 can be considered as *O*(3*kn*). The third step is the Region-Merging process. Suppose that *r*_1_ is the number of regions before merging and that *r*_2_ is the number of regions merged. The complexity of calculating the difference between regions is $O({3r}_{1}^{2})$, and the complexity of merging the regions is *O*(3*r*_2_*n*). Therefore, the complexity of step 3 is $O(3{(r_{1}^{2}+r}_{2}n))$. To summarize, the expected time complexity of the *AHHT* algorithm is $O(3{(kn+r_{1}^{2}+r}_{2}n))$. It is worth mentioning that the histon-based and roughness-index-based algorithms need to calculate the Euclidean distance 24*n* times to find the thresholds. By contrast, the *AHHT* algorithm has substantially reduced the time consumption.

## Experimental results

As two variations of histogram-based techniques, the histon-based and roughness-index-based techniques have been demonstrated to achieve better segmentation results. In this study, the performance of the proposed *AHHT* technique is compared with them. The experiments are performed on Berkeley Segmentation Data Set 300 (BSDS300) as well as Berkeley Segmentation Data Set 500 (BSDS500). Each image is 481 × 321 pixels. For each image, a set of ground truths compiled by the human observers is provided. All the images are normalized to have the longest side equivalent to 320 pixels.

All of these techniques include three major steps, and each one of the steps offers similar functionality. For the histon-based and roughness-index-based techniques, all parameters involved are set the same as those used in the original papers [31,33]. Concretely, in step 1, two parameters are involved for finding the significant peaks: (1) the peak is greater than 20% of the average value of all peaks; and (2) the distance between two peaks is greater than 10. In the post-processing step (step 3), two parameters *T*_*n*_ and *T*_*d*_ for region merging are involved. Unless otherwise stated the results, *T*_*n*_ is set as 0.1%, and *T*_*d*_ is set as 20, respectively. For the proposed *AHHT* algorithm, only one parameter, the bin merge threshold *w*, is involved in step 1. In our experiments, *w* is set as 15, which means that any adjacent pair of bins cannot be merged if the difference between the two bins is larger than 15. In the same post-processing step, the two involved parameters are identical to those of the histon-based and roughness-index-based algorithms to make a fair comparison.

### Visual evaluation of segmentation results

**Fig 4:**
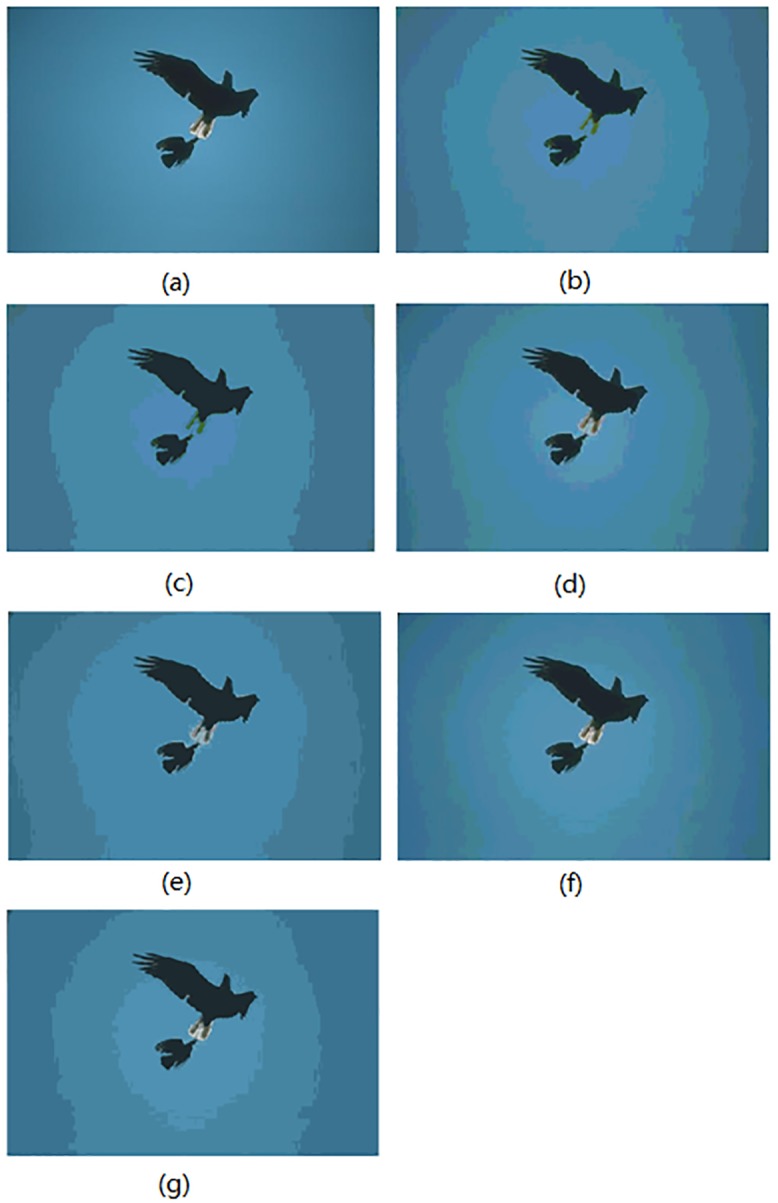
The image *Birds*: (a) original image, (b, c) initial segmented result and result after region merging based on the histon, (d, e) initial segmented result and result after region merging based on the roughness index, (f, g) initial segmented result and result after region merging based on *AHHT*.

**Fig 5:**
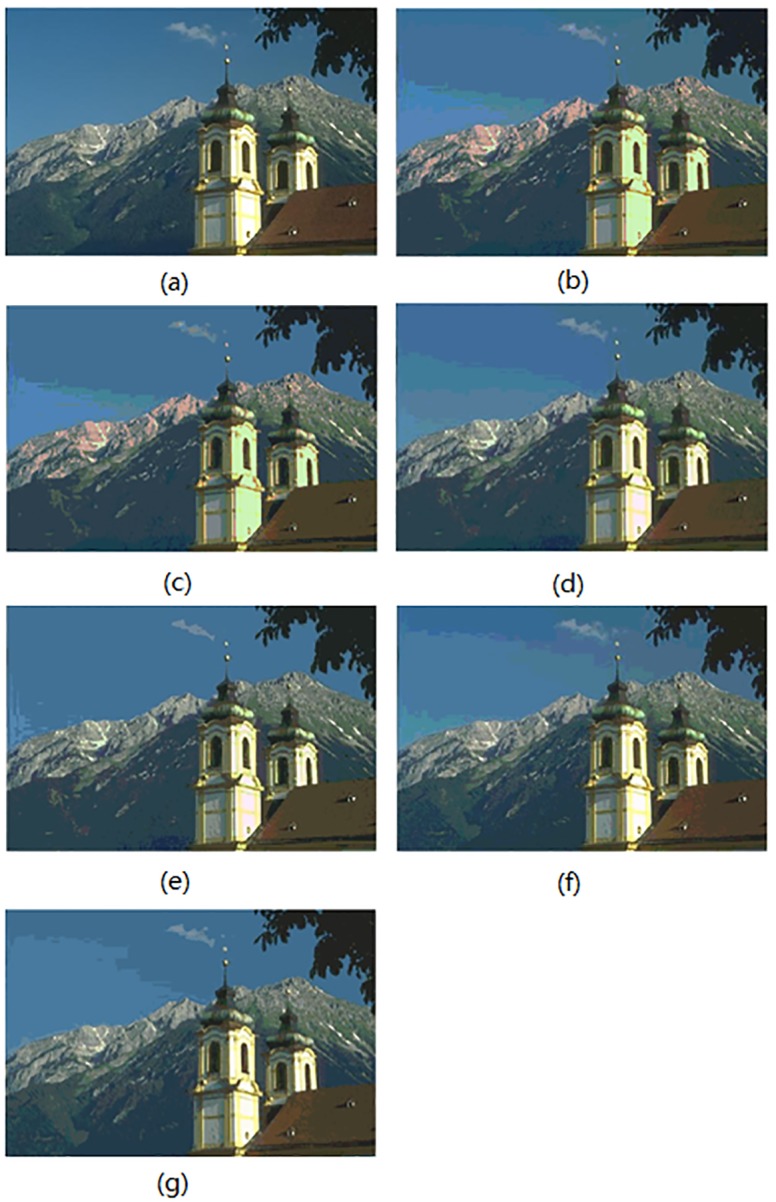
The image *Church*: (a) original image, (b, c) initial segmented result and result after region merging based on the histon, (d, e) initial segmented result and result after region merging based on the roughness index, (f, g) initial segmented result and result after region merging based on *AHHT*.

**Fig 6:**
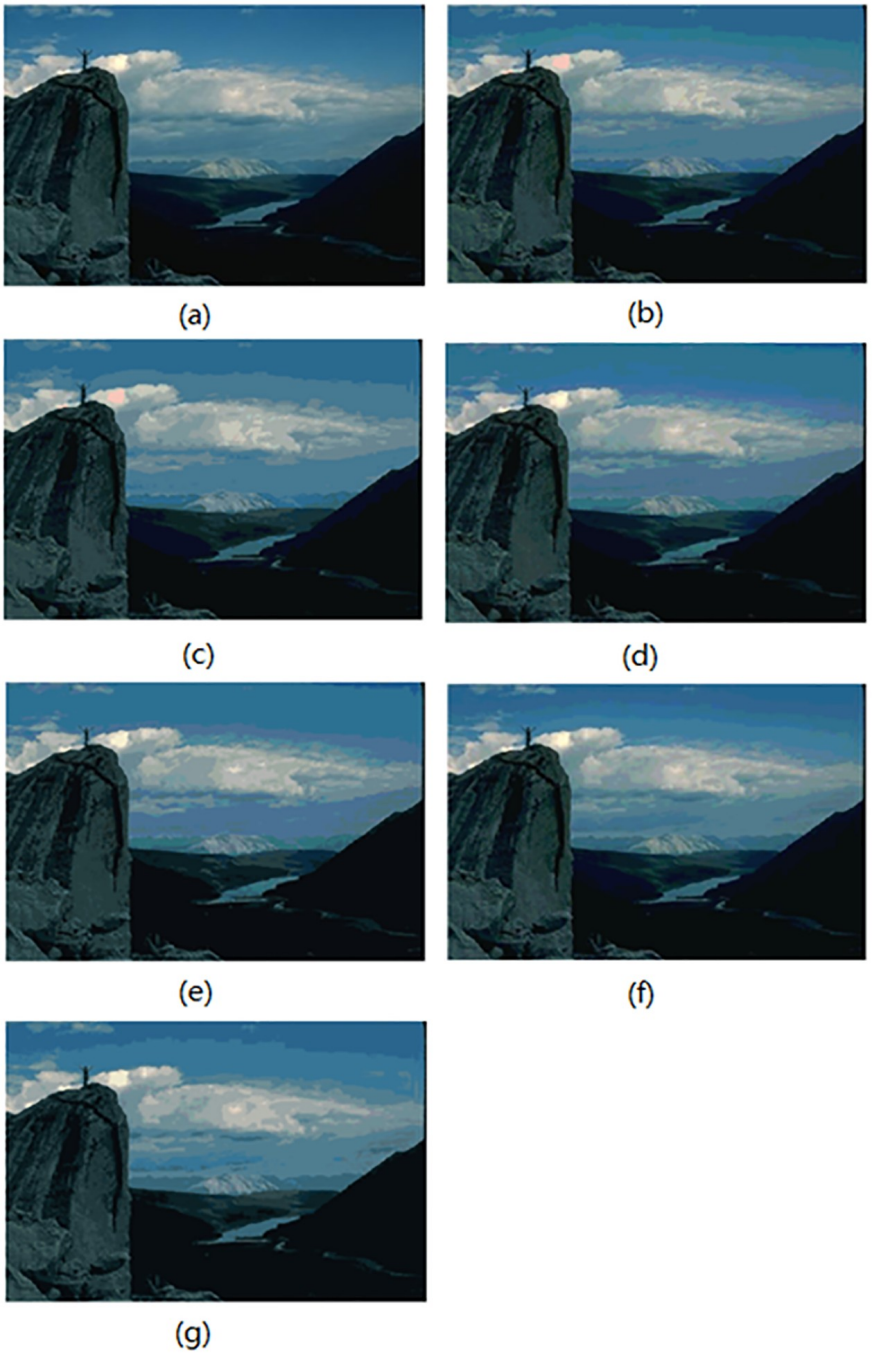
The image *Mountain*: (a) original image, (b, c) initial segmented result and result after region merging based on the histon, (d, e) initial segmented result and result after region merging based on the roughness index, (f, g) initial segmented result and result after region merging based on *AHHT*.

**Fig 7:**
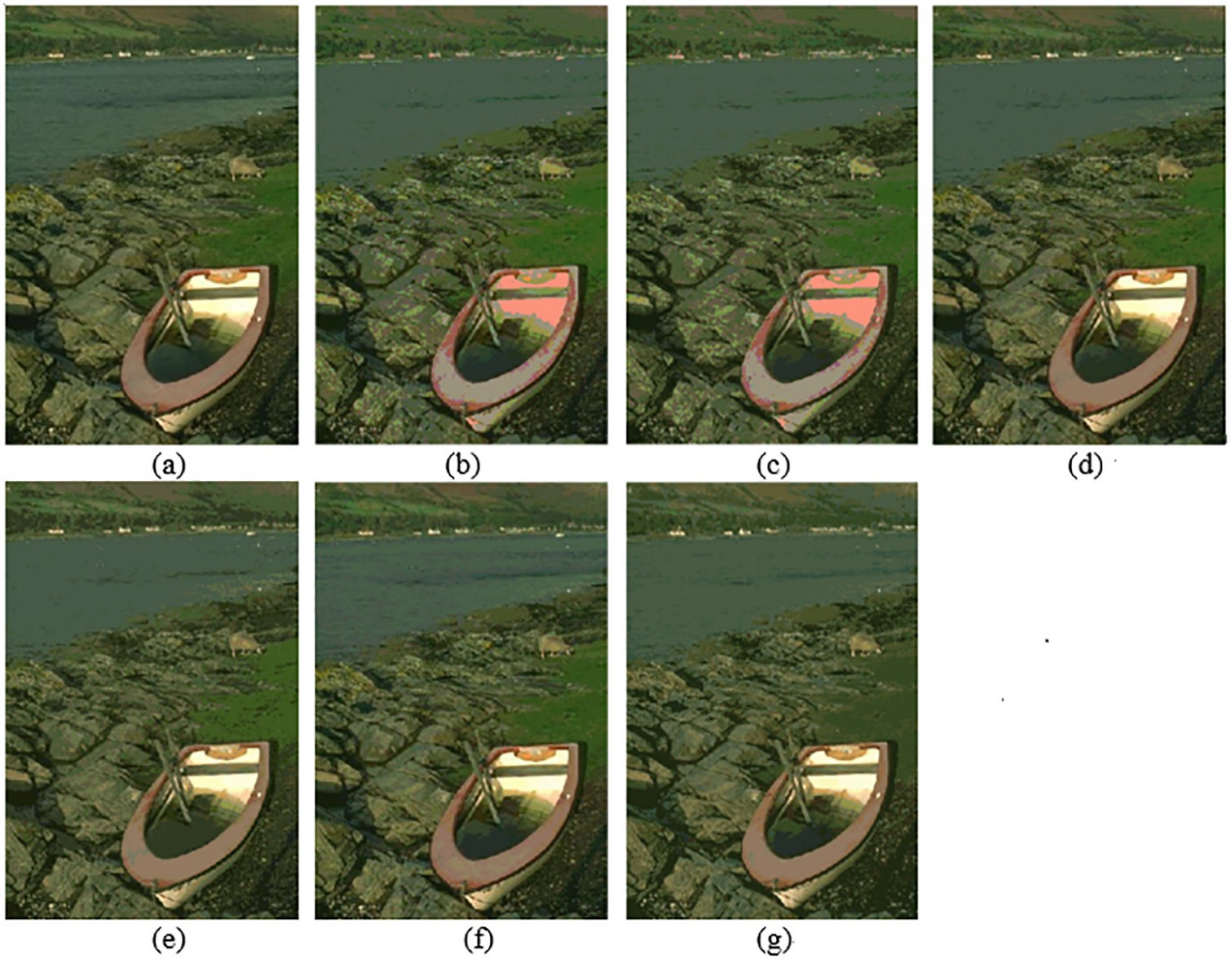
The image *Marsh*: (a) original image, (b, c) initial segmented result and result after region merging based on the histon, (d, e) initial segmented result and result after region merging based on the roughness index, (f, g) initial segmented result and result after region merging based on *AHHT*.

**Fig 8:**
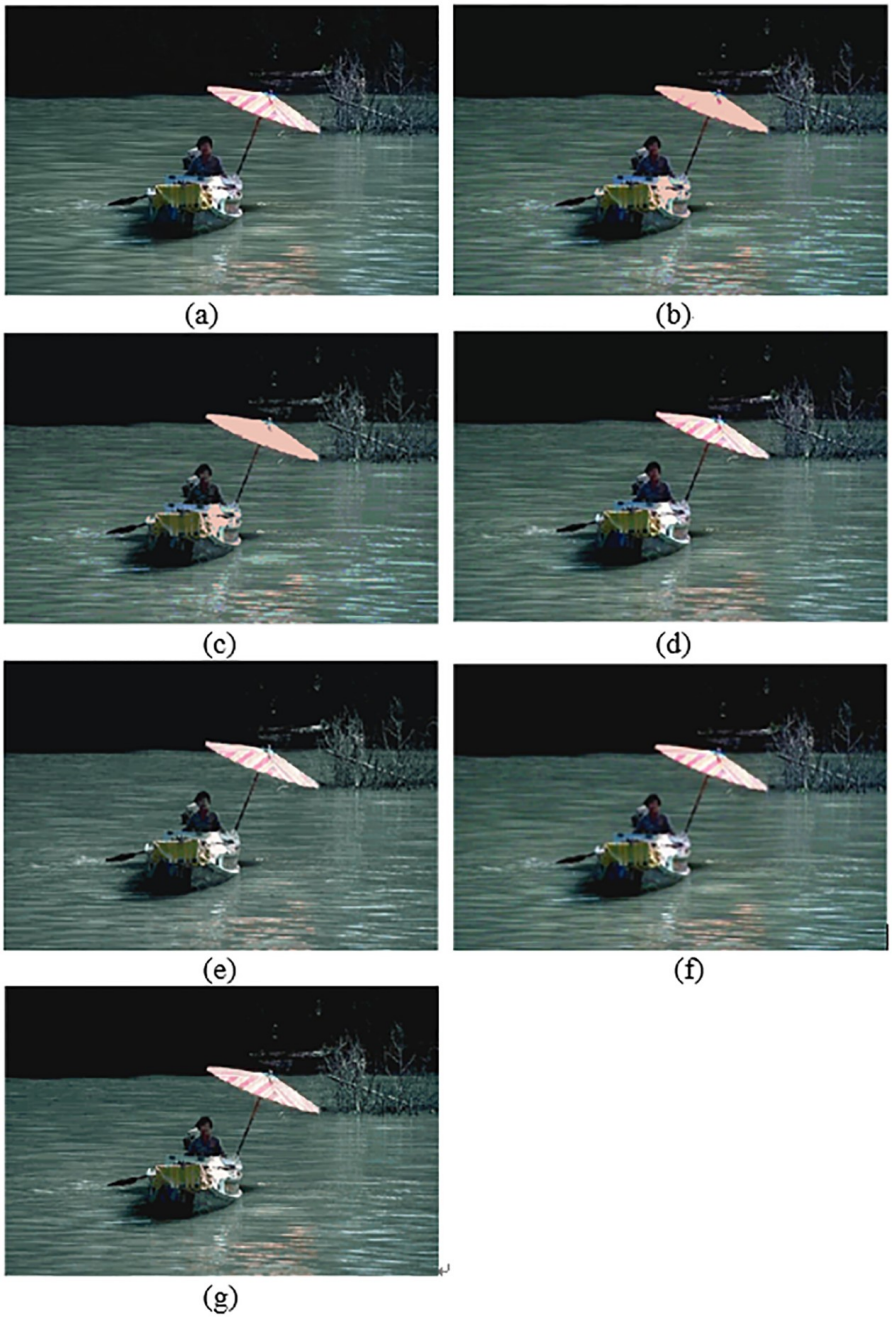
The image *Boating*: (a) original image, (b, c) initial segmented result and result after region merging based on the histon, (d, e) initial segmented result and result after region merging based on the roughness index, (f, g) initial segmented result and result after region merging based on *AHHT*.

**Fig 9:**
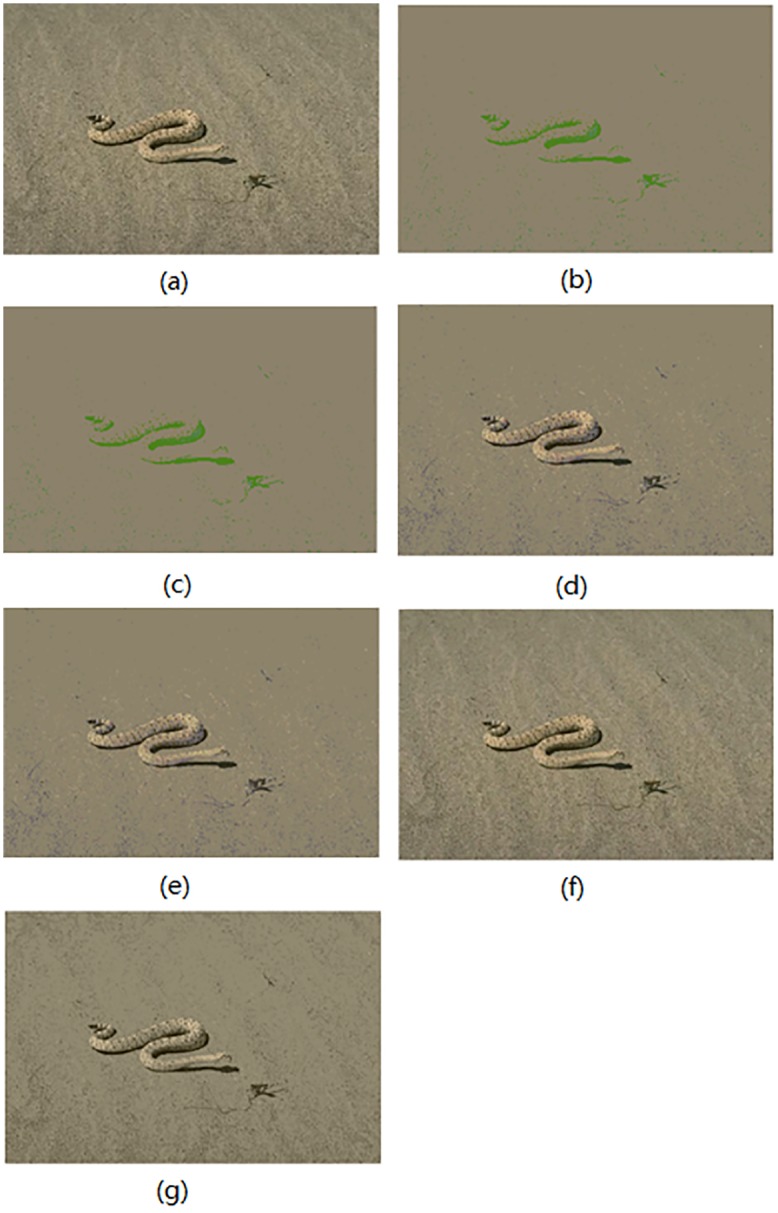
The image *Snake*: (a) original image, (b, c) initial segmented result and result after region merging based on the histon, (d, e) initial segmented result and result after region merging based on the roughness index, (f, g) initial segmented result and result after region merging based on *AHHT*.

In this section, the segmentation results for compared algorithms are visually evaluated by using 6 of all the segmented images. The segmentation results for the images *Birds(*#135069), *Church(*#126007), *Mountain*(#14037), *Marsh*(#92059), *Boating*(#147021) and *Snake*(#196073) are shown in Figs 4–9, respectively. Considering that all of the compared techniques adopt the same *Region-Merging* processing, in Figs 4–9, we present the initial segmented result and the result after region merging for each technique. In Table 1, columns 3–5 present the number of bands in each plane of *R*, *G*, and *B* of the initial segmented result, and columns 6–7 present the color number in the initial segmented result and the color number in the postmerging result. Generally, based on visual evaluation, the *AHHT* technique produces better segmentation results.

**Table 1:** Comparison of the number of thresholds and the color number in the initial segmented result and the result after merging.

*Image*	Methods	Red band	Green band	Blue band	Color number	
					Initial segmentation	After merging
Birds #135069	Histon	3	5	6	18	6
	Roughness-index	8	11	13	73	10
	AHHT	41	23	17	308	13
Church #126007	Histon	8	8	8	210	36
	Roughness-index	12	9	14	245	35
	AHHT	13	11	15	274	40
Mountain #14037	Histon	12	13	9	128	22
	Roughness-index	15	15	15	201	27
	AHHT	21	19	15	181	22
Marsh #92059	Histon	10	5	6	125	29
	Roughness-index	17	13	17	323	31
	AHHT	13	15	14	245	33
Boating #147021	Histon	11	10	10	290	27
	Roughness-index	14	17	17	373	35
	AHHT	13	15	16	322	46
Snake #196073	Histon	2	3	4	10	10
	Roughness-index	9	9	7	35	17
	AHHT	25	25	23	197	11

For the image *Birds*, Fig 4 shows the initial segmented result and the result after region merging for the histon-based, roughness-index-based and *AHHT* techniques. For the histon, roughness-index- and *AHHT* techniques, the numbers of colors in the initial segmented results are 18 (Fig 4b), 73 (Fig 4d) and 308 (Fig 4f), respectively; the numbers of colors in the final segmented results are 6 (Fig 4c), 10 (Fig 4e) and 13 (Fig 4g), respectively. For the histon-based technique (Fig 4b and 4c), we can see that there are fewer colors in the segmented results, which leads to larger homogenous regions in the results. However, the white feathers of the birds have been mistakenly assigned to the sky by the histon technique. For the roughness-index-based technique (Fig 4d and 4e), the white feathers of the birds have been assigned to a color close to that of the sky. By contrast, the *AHHT* technique has successfully avoided this classification error. Therefore, although the initial segmented results based on the histon and roughness index produced a lower number of colors, they lose many details of small distinct regions. It is worth noting that, although all three techniques adopt the same region merging process, the number of colors in the final segmented result by the histon technique is obviously less than that of the other two techniques. The reason for this is that, for the histon technique, there are small color differences between the different regions in the initial segmented result, which in turn cause these different regions to be further merged.

For the image *Church*, Fig 5 shows the initial segmented result and the result after region merging for the histon-based, roughness-index-based and *AHHT* techniques, respectively. For the histon-based, roughness-index-based and *AHHT* techniques, the numbers of colors in the initial segmented result are 210 (Fig 5b), 245 (Fig 5d) and 274 (Fig 5f), respectively; the numbers of colors in the final segmented results are 36 (Fig 5c), 35 (Fig 5e) and 40 (Fig 5g), respectively. In the histon-based segmentation of Fig 5b and 5c, the red mountain ridge in the distance and the light green of the exterior wall of the building do not match with those in the original image. In addition, in the histon-based and roughness-index-based segmentations of Fig 5b–5e, we observed that the very dark color of the gentle mountain slope at the left bottom corner does not match those in the original image. One can see from the original image (Fig 5a) that there is a clearly green boundary between the gentle mountain slope at the left bottom corner and the middle mountain. However, this green boundary is almost gone in the segmented results (Fig 5b–5e). Whereas in the *AHHT* technique of segmentation, as shown in Fig 5f and 5g, we observe that the colors of buildings, mountains, the sky and clouds match exactly with colors of the corresponding regions in the original image.

For the image *Mountain*, Fig 6 shows the initial segmented result and the result after region merging for the histon-based, roughness-index-based and *AHHT* techniques. For the histon-based, roughness-index-based and *AHHT* techniques, the numbers of colors in the initial segmented result are 128 (Fig 6b), 201 (Fig 6d) and 181 (Fig 6f), respectively; the numbers of colors in the final segmented results are 22 (Fig 6c), 27(Fig 6e) and 22 (Fig 6g), respectively. In the histon-based segmentation of Fig 6b and 6c, the color of the distant mountain in the middle of the image is a slight violet, and the color of the cloud on the top of the statue is assigned to the color of pink, which do not match those in the original image. For the roughness-index-based segmentations of Fig 6d and 6e, the color of the middle sky is violet, and the color of the distant mountain is assigned to the color of the top sky, which also do not match those in the original image. However, the *AHHT* technique prevents the above classification errors. We can see from Fig 6f and 6g that the *AHHT* technique yields much better segmentation results, results in which the colors of clouds, the sky and the distant mountains match those in the original image.

For the image *Marsh*, Fig 7 shows the initial segmented result and the result after region merging for the histon-based, roughness-index-based and *AHHT* techniques, respectively. For the histon-based, roughness-index-based and *AHHT* techniques, the numbers of colors in initial segmented result are 125 (Fig 7b), 323 (Fig 7d) and 245 (Fig 7f), respectively; and the numbers of colors in final segmented results are 29 (Fig 7c), 31(Fig 7e) and 33 (Fig 7g), respectively. In histon-based segmentation of Fig 7b and 7, we can see that the color of the inner surface is pink, which does not match the white color of the corresponding regions in the original image. In addition, although both the histon-based and roughness-index-based techniques produce more homogenous water surface, there are considerable pixels of the nearshore that are assigned as part of the water surface. By contrast, in the segmented results (Fig 7f and 7g) of the *AHHT* technique, the border between the water surface and the nearshore is nicely retained. As shown in Fig 7f and 7g, we observe that the colors of the boat, water surface, and nearshore match the colors of the corresponding regions in the original image.

For the image *Boating*, Fig 8 shows the initial segmented result and the result after region merging for the histon-based, roughness-index-based and *AHHT* techniques. For the histon-based, roughness-index-based and *AHHT* techniques, the numbers of colors in the initial segmented result are 290 (Fig 8b), 373 (Fig 8d) and 322 (Fig 8f), respectively; the numbers of colors in the final segmented results are 27 (Fig 8c), 35(Fig 8e) and 46 (Fig 8g), respectively. In the histon-based segmentation of Fig 8b and 8c, we can see the red and yellow on the top of the umbrella and the white on the side of the bow, and in the original image, these are changed to pink. In the roughness-index-based segmentation of Fig 8d and 8e, the yellow and green stripe on the surface of the bow is causing a slight confusion. By contrast, the *AHHT* technique obtains better segmentation results; see Fig 8f and 8g.

For the image *Snake*, Fig 9 shows the initial segmented result and the result after region merging for all comparison techniques. For the histon-based, roughness-index-based and *AHHT* techniques, the numbers of colors in the initial segmented result are 10 (Fig 9b), 35 (Fig 9d) and 197 (Fig 9f), respectively; and the numbers of colors in the final segmented results are 5 (Fig 9c), 17 (Fig 9e) and 11 (Fig 9g), respectively. In histon-based segmentation of Fig 9b and 9c, we can see that there are fewer colors in the segmented results, which leads to larger homogenous regions in the results. However, the colors of the snake and desert are the same color, and they do not match their colors in the original image. In addition, the color of the snake′s shadow is seen as light green instead of black. In case of roughness-index-based segmentation of Fig 9d and 9e, the texture of sand surface is not clearly visible, although the snake is clearly visible. In contrast, we can see from the *AHHT*-based segmentation of Fig 9f and 9g that the colors of the snake, the snake′s shadow and the desert, and the texture of the sand surface match those in the original image.

The analysis of the above experiments illustrates that the initial segmentation plays a decisive role in the full process of segmentation. *AHHT* obtains the best visual features in the initial segmentation, which in turn allows it to produce the best visual features in the end.

In addition, to better analyze the characteristics of the compared techniques, Table 2 presents the mean number of colors in the initial segmented results and the results after region merging over all images of the BSDS300 and the BSDS500. Take BSDS500 for example, for the histon-based, roughness-index-based and *AHHT* techniques, the mean numbers of colors in the initial segmented results are 338, 377 and 333, respectively; the mean number of colors in the final segmented results are 44, 47 and 49, respectively. This illustrates that, although all compared techniques adopt the same *Region-Merging* processing, the *AHHT* technique obtains a slightly larger number of colors in the final segmented results. The reason for this is that, for the *AHHT* technique, there are relatively larger differences between the colors in the initial segmented result, which in turn restricts those colors from being further merged, thus ensuring good segmentation quality.

**Table 2:** Comparison between the mean number of colors in the initial segmented result and the result after merging.

Image	Methods	Mean number of colors		Image	Methods	Mean number of colors	
		Initial segmentation	After merging			Initial segmentation	After merging
BSDS300	Histon	316	41	BSDS500	Histon	338	44
	Roughness-index	355	42		Roughness-index	377	47
	AHHT	327	46		AHHT	333	49

### Quantitative evaluation of segmentation results

In this section, the results of each image segmentation technique are compared using quantitative evaluations, such as the mean square error (*MSE*), *F*(*I*) [37], and *Q*(*I*) [38]. The *MSE* evaluation function can be described as

$$MSE=\frac{1}{M\times N}\sum_{m=1}^{M}\sum_{n=1}^{N}\sum_{i\in \left\{R,G,B\right\}}{\left(I(m,n,i)-I^{\prime }(m,n,i)\right)}^{2},$$ (10)

where *I* is the original *RGB* color image, *M* × *N* is the image size, and *I*′ is the segmented image of *I*. In general, a lower *MSE* indicates good segmentation quality of the output in the case that the numbers of regions are close for different segmented results. The evaluation function of *F*(*I*) is defined as follows [37]:

$$F(I)=\frac{1}{1000(M\times N)}\sqrt{R}\sum_{j=1}^{R}\frac{e_{j}^{2}}{\sqrt{A_{j}}},$$ (11)

and *Q*(*I*) is further refined from *F*(*I*) by Borsotti *et al*. as follows [38]:

$$Q(I)=\frac{1}{1000(M\times N)}\sqrt{R}\sum_{j=1}^{R}\left[\frac{e_{j}^{2}}{1+logA_{j}}+{\left(\frac{S(A_{j})}{A_{j}}\right)}^{2}\right],$$ (12)

where *I* is the segmented color image of size *M* × *N*, *R* is the number of regions of the segmented image, *A*_*j*_ denotes the number of pixels in the *j*th region. *e*_*j*_ is defined as the sum of the color differences between the *RGB* color vectors of the pixels of the *j*th region and the color vector attributed to the *j*th region, and *S*(*A*_*j*_) represents the number of regions having an area equal to *A*_*j*_. Although *F*(*I*) and *Q*(*I*) are different, both measures are used to penalize segmentations that form too many regions and have nonhomogeneous regions by assigning them larger values.

The *MSE*, *F*(*I*) and *Q*(*I*) values of segmentation results are tabulated in Table 3 for the images shown in Figs 4–9. The smaller the values of these indexes, the better the segmentation result should be. The bolded values indicate the best results. The comparison results show that the *AHHT* technique obtains the best *MSE*, *F*(*I*) and *Q*(*I*) values on the same three images; the roughness-index-based technique obtains the best *F*(*I*) and *Q*(*I*) values on the one image. The direct comparison of these results can be obtained by checking the mean value in the last row in Table 3. Obviously, the *AHHT* technique outperforms the roughness-index-based and histon-based techniques by obtaining the relatively small mean values of indexes in segmenting all of these images.

**Table 3:** Comparison *MSE*, *F*(*I*) and *Q*(*I*) evaluation function of segmentation results.

Image	MSE			F(I)			*Q(I)*		
	Histon	Roughness-index	AHHT	Histon	Roughness-index	AHHT	Histon	Roughness-index	AHHT
Birds	11.59010	11.53233	**8.61797**	52.67094	70.64469	**42.93231**	78.72281	109.90817	**65.06391**
Church	14.80610	13.21823	**12.02938**	105.59323	73.15529	**72.32908**	74.04804	**62.60939**	69.32062
Mountain	10.39769	9.93918	**8.95015**	44.98419	43.65162	**31.06930**	39.11403	38.92950	**27.31039**
Marsh	14.51536	12.17336	**11.83566**	105.72585	77.40083	**74.04451**	84.94691	**74.75858**	75.07219
Boating	11.00687	9.32096	**9.06405**	76.88258	**42.72762**	50.86452	56.00279	**37.20927**	49.51961
Snake	17.67190	13.49228	**9.59335**	184.02131	127.79234	**52.51785**	338.82650	233.31171	**82.90309**
**Mean value**	13.33134	11.61272	**10.01509**	94.97968	72.56207	**53.95959**	111.94351	92.78777	**61.53164**

To better support the abovementioned findings, the mean values of *MSE*, *F*(*I*) and *Q*(*I*) are tabulated in Table 4 for all images of the BSDS300 and images of the BSDS500. From the results in Table 4, it is clear that the proposed *AHHT* technique outperforms the other techniques based on the *MSE*, *F*(*I*) and *Q*(*I*) measures.

**Table 4:** Comparison average of *MSE*, *F*(*I*) and *Q*(*I*) on BSDS300 and on BSDS500.

Image	Methods	MSE (Mean)	F(I) (Mean)	Q(I) (Mean)	Image	Methods	MSE (Mean)	F(I) (Mean)	*Q(I) (Mean)*
BSDS300	Histon	12.13341	69.86678	62.92299	BSDS500	Histon	12.09972	68.85657	61.05272
	Roughness-index	11.69978	70.19091	65.72476		Roughness-index	11.70673	68.25779	61.58926
	AHHT	**10.97841**	**57.7446**	**53.18803**		AHHT	**11.04783**	**57.97239**	**51.83581**

The above benchmark indices are used to estimate the empirical accuracy of the segmentation results. They include some human characterizations on the properties of ideal segmentation requiring no prior knowledge of correct segmentation.

For each image in the BSDS, a set of ground truths compiled by human observers is provided. Therefore, we intend to compare segmentation results against external criteria. The following image segmentation indices were used. The Probability Rand Index (PRI) counts the fraction of pairs of pixels whose labels are consistent between the computed segmentation and the ground truth, averaging across multiple ground truth segmentations to account for scale variation in human perception [39]. The Variation of Information (VOI) is used for quantification of the loss of information and the gain between two clusters belonging to the lattice of possible partitions [40]. The Boundary Displacement Error (BDE) is used for evaluation of the average displacement error of boundary pixels between two segmented images by computing the distance between the pixel and the closest pixel in the other segmentation [41]. The Global Consistency Error (GCE) is used for quantification of the extent to which a segmentation can be viewed as the refinement of the others [42]. These four measures must be considered all together to evaluate the performance of a given segmentation algorithm. Higher values of PRI indicate a large similarity between the segmented images and the ground truth; whereas for rest of the indices, lower values indicate closer similarity of the segmentation obtained and the ground truth.

Table 5 presents the average performance indices (PRI, BDE, GCE and VOI) obtained by the proposed AHHT algorithm compared with Histon and Roughness-index algorithms. As mentioned above, these three algorithms are histogram-based algorithms. In addition, Table 5 also lists results of some other popular algorithms. The results of Mean-Shift [43], NCuts [44], FH [45], CTM [12], and MCET_DE [27], were obtained from literature sources [12,27]. For the three histogram-based techniques of Histon, Roughness-index, and AHHT, we found an improvement in the results in terms of GCE and VOI with a larger value of *T*_*d*_, in contrast to that of the PRI measurement, which decreased. Compared with Histon and Roughness-index algorithms, the AHHT algorithm obtains better values of BDE, GCE and VOI when the same value of *T*_*d*_ is used. From Table 5, it can be seen that the three histogram-based techniques of Histon, Roughness-index, and AHHT can obtain superior BDE values compared with other algorithms.

**Table 5:** Comparison averages of PRI, BDE, GCE and VOI on BSDS300.

Algorithm	PRI	BDE	GCE	VOI
Mean-Shift	0.7550	9.7001	0.2598	2.4770
Ncuts	0.7229	9.6038	0.2182	2.9329
FH	0.7841	9.9497	0.1895	2.6447
CTM(n = 0.2)	0.7617	9.8962	0.1877	2.0236
MCET_DE(Q = 15,LV = 7)	0.7493	9.6597	0.2542	2.1864
Histon(*T*_*d*_ = 20)	0.72361	9.476	0.41158	4.246
Histon(*T*_*d*_ = 70)	0.63756	9.1357	0.30279	2.6513
Roughness-index(*T*_*d*_ = 20)	0.72333	9.4655	0.41292	4.226
Roughness-index(*T*_*d*_ = 70)	0.6396	8.7628	0.30521	2.6578
AHHT(*T*_*d*_ = 30)	0.71937	9.1668	0.43263	3.4961
AHHT(*T*_*d*_ = 70)	0.63414	8.6678	0.29859	2.6386

### Runtime comparison

The computational efficiency of the algorithm is a key factor that imposes a large influence upon its practical application. In this section, the efficiencies of the three techniques are compared as the execution time in seconds. Considering that the execution times for all compared techniques include two parts, Table 6 presents the mean time spent on the initial segmentation, the mean time spent on the Region-Merging process, and the mean total time (the sum of the two former) spent on BSDS300 as well as BSDS500.

**Table 6:** Mean execution time (in seconds) of different algorithms.

Image	Histon			Roughness-index			AHHT		
	Initial segmentation	Merging process	Total	Initial segmentation	Merging process	Total	Initial segmentation	Merging process	Total
BSDS300	8.1459	2.0008	10.14673	9.1763	2.7703	11.9467	**0.0685**	**2.4703**	**2.5388**
BSDS500	9.8467	2.6347	12.4814	8.3534	2.8033	11.1567	**0.0681**	**2.2625**	**2.3307**

From Table 6, we can see that the mean time of initial segmentation of an image is approximately 9 seconds for the histon-based and roughness-index-based techniques. By contrast, the time of initial segmentation of an image is approximately 0.07 seconds for the *AHHT* technique, which means that *AHHT* outperforms the histon-based and roughness-index-based techniques by up to two orders of magnitude in the matter of efficiency of initial segmentation. The complexity analysis shows that the major reason for the big difference is the time required to find the thresholds: the histon-based and roughness-index-based techniques need to calculate the Euclidean distance 24*n* times; however, the *AHHT* technique mainly needs 3*n* instances of pixel access with no complicated calculation involved. Therefore, the *AHHT* technique obtains the great advantage of efficiency in initial segmentation.

The time spent on the *Region-Merging* process mainly depends on the number of merged regions. In this process, the differences between the compared techniques are not noticeable. The full execution time to segment an image mainly depends on the initial segmentation for the histon-based and roughness-index-based techniques. In contrast, for the *AHHT* algorithm, the full execution time to segment an image largely depends on the merging process. From Table 6, we can see that the *AHHT* technique obtains significantly faster running speeds.

## Conclusion

This paper presents a novel histogram thresholding—Adaptive Hierarchical-Histogram Thresholding (AHHT), which is an adaptive thresholding algorithm used to perform color image segmentation. The contributions of the paper include the following. (1) A structure called *hierarchical-histogram* has been proposed in the paper. With the aid of *hierarchical-histogram*, the *AHHT* algorithm can adaptively identify the thresholds at valleys. (2) *AHHT* does not need to find the significant peaks. (3) The experimental results show that the *AHHT* algorithm can obtain better results for color image segmentation. (4) For the simplicity of implementation, the *AHHT* algorithm has fast running speed. The experimental results show that *AHHT* outperforms the compared algorithms by up to two orders of magnitude in the matter of efficiency of initial segmentation.

## Supporting information

S1 File Includes the results of AHHT (Td = 20) on each image in BSDS300. (XLS)

S2 File Includes the results of AHHT (Td = 20) on each image in BSDS500. (XLS)

S3 File Includes the results of AHHT (Td = 30) on each image in BSDS300. (XLS)

S4 File Includes the results of Histon (Td = 20) on each image in BSDS300. (XLS)

S5 File Includes the results of Histon (Td = 20) on each image in BSDS500. (XLS)

S6 File Includes the results of PRI,BDE, GCE and VOI. (XLSX)

S7 File Includes the results of Roughness(Td = 20) on each image in BSDS300. (XLS)

S8 File Includes the results of Roughness(Td = 20) on each image in BSDS500. (XLS)

S1 Data Includes the segmented results of AHHT (Td = 30) on each image in BSDS300. The folder being used for calculating the metrics of PRI,BDE, GCE and VOI.
(ZIP)

S2 Data Includes the segmented results of AHHT (Td = 70) on each image in BSDS300, the folder being used for calculating the metrics of PRI,BDE, GCE and VOI. (ZIP)

S3 Data Includes the segmented results of Histon (Td = 20) on each image in BSDS300, the folder being used for calculating the metrics of PRI,BDE, GCE and VOI. (ZIP)

S4 Data Includes the segmented results of Histon (Td = 70) on each image in BSDS300, the folder being used for calculating the metrics of PRI,BDE, GCE and VOI. (ZIP)

S5 Data Includes the segmented results of Roughness(Td = 20) on each image in BSDS300, the folder being used for calculating the metrics of PRI,BDE, GCE and VOI. (ZIP)

S6 Data Includes the segmented results of Roughness(Td = 70) on each image in BSDS300, the folder being used for calculating the metrics of PRI,BDE, GCE and VOI. (ZIP)
